# Supplementary material for: Imbalance in Unc80 RNA Editing Disrupts Dynamic Neuronal Activity and Olfactory Perception
Source: Int J Mol Sci. 2024 May 30;25(11):5985. doi: 10.3390/ijms25115985 (PMC11172567; doi:10.3390/ijms25115985)
Supplement: Supplementary file 1 [file ijms-25-05985-s001.zip › Supplementary Figure.pdf]

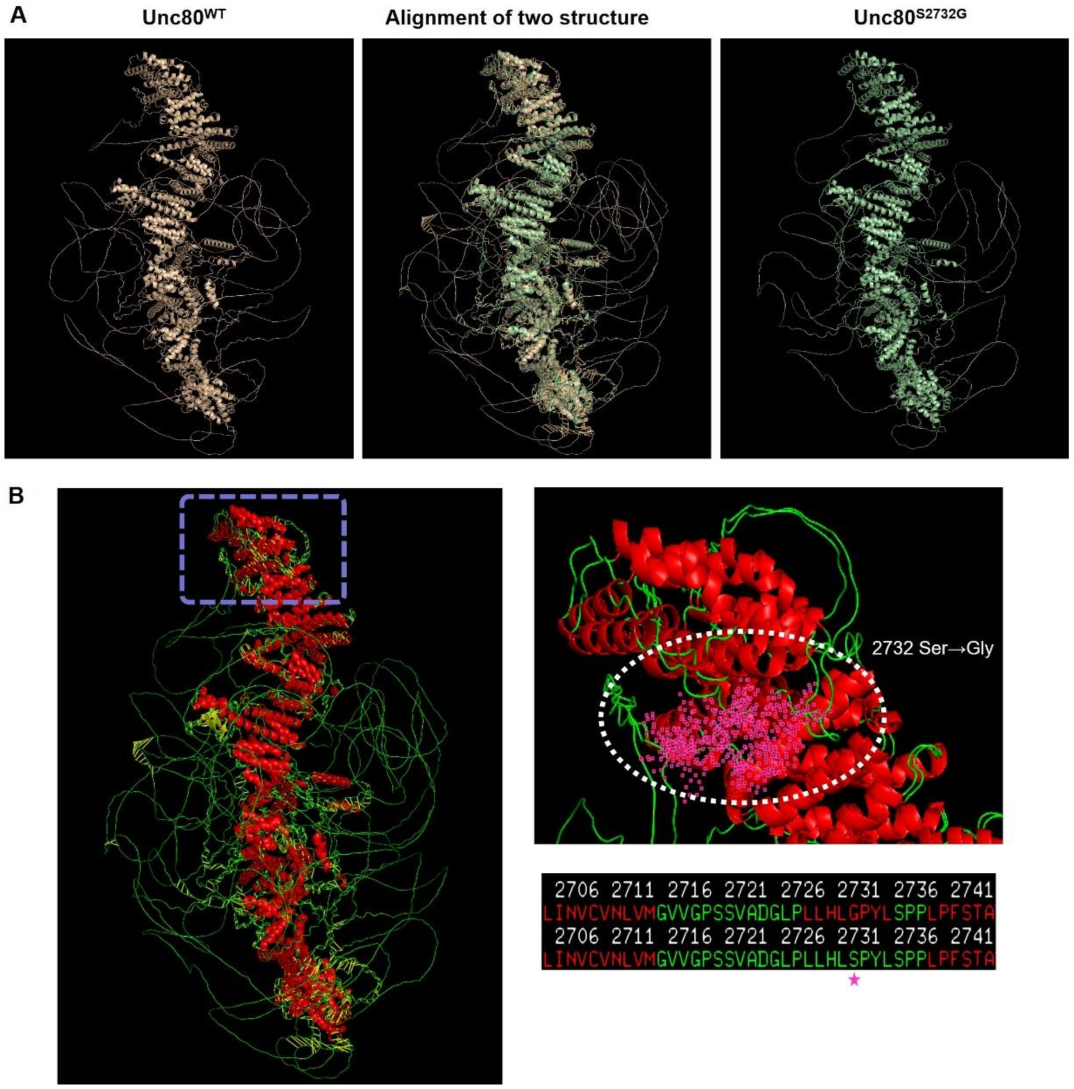


**Supplementary Figure S1. AlphaFold2 structure prediction of Unc80$^{S2732G}$ compared to Unc80$^{WT}$.** (**A**) Full-length models of Unc80$^{WT}$ and of Unc80$^{S2732G}$, both composed of 3326 amino acid residues, predicted by AlphaFold2. The left and right panels respectively show the structures of Unc80$^{WT}$ and of Unc80$^{S2732G}$, while the middle panel displays the alignment of Unc80$^{WT}$ and Unc80$^{S2732G}$. (**B**) The structural sub-region encompassing the recoded amino acid of Unc80 at residue 2732 is marked with the dotted box (left panel), with the magnified view shown in the right panel. Dotted circle outlines key residues undergoing recoding-driven conformational change in the Unc80$^{WT}$ structure. Bottom right panel: primary sequence alignment of the wild-type (bottom) and edited sequences (amino acid substitution marked by purple star). Red letters denote sequence regions in alpha-helical conformation, whereas green letters denote non-structured sequences.

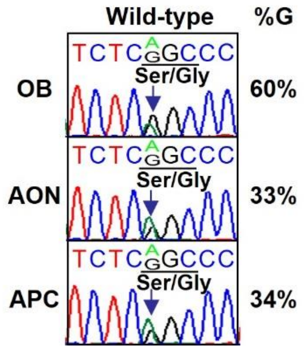


**Supplementary Figure S2. RNA editing level of *Unc80* in OB, AON, and APC.** Total RNA was extracted from the indicated brain regions from wild-type mice, then subjected to PCR and Sanger sequencing. The representative sequence chromatograms display the editing level at the A-to-G editing site of Unc80 in the indicated regions. The percentage represents the editing frequency (%G), as calculated in Figure 1. (OB: olfactory bulb, AON: anterior olfactory nucleus, APC: anterior piriform cortex.)

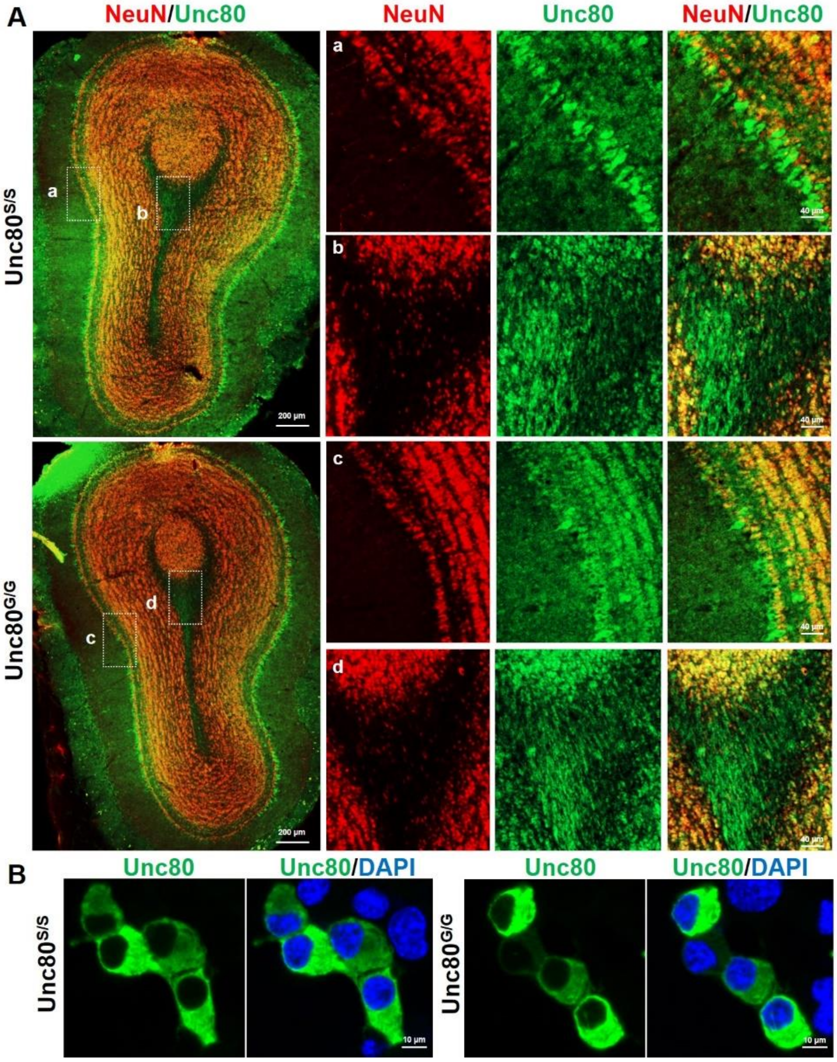


**Supplementary Figure S3. Localization of Unc80 in the Unc80$^{S/S}$ and Unc80$^{G/G}$ knock-in mice and N2a cells. (A)** Coronal sections of olfactory bulb from Unc80$^{S/S}$ and Unc80$^{G/G}$ knock-in mice immunostained with NeuN antibody (red) and Unc80 antibody (green). Scale bars = 200 µm in the left panel and 40 µm in the magnified images (a to d). (**B**) Neuron 2a cells were transfected with specific GFP-tagged wild-type Unc80 or GFP-tagged Unc80$^{S2732G}$ encoding plasmids for 24hr, then fixed and co-stained with DAPI (nuclear staining). The images were analyzed for GFP signal (green) by confocal microscopy. Scale bars = 10 µm.
